# Stepwise approach to SNP-set analysis illustrated with the Metabochip and colorectal cancer in Japanese Americans of the Multiethnic Cohort

**DOI:** 10.1186/s12864-018-4910-8

**Published:** 2018-07-09

**Authors:** John Cologne, Lenora Loo, Yurii B. Shvetsov, Munechika Misumi, Philip Lin, Christopher A. Haiman, Lynne R. Wilkens, Loïc Le Marchand

**Affiliations:** 1 Department of Statistics, Radiation Effects Research Foundation, Hiroshima, 732-0815 Japan; 2 Epidemiology Program, University of Hawaii Cancer Center, Honolulu, HI 96813 USA; 3 Department of Preventive Medicine and Norris Comprehensive Cancer Center, Keck School of Medicine, University of Southern California, Los Angeles, CA 90033 USA; 4 Biostatistics and Informatics Shared Resource, University of Hawaii Cancer Center, Honolulu, HI 96813 USA

**Keywords:** Colorectal cancer, Genome-wide association study, Metabochip array, Multiethnic Cohort, SNP set analysis

## Abstract

**Background:**

Common variants have explained less than the amount of heritability expected for complex diseases, which has led to interest in less-common variants and more powerful approaches to the analysis of whole-genome scans. Because of low frequency (low statistical power), less-common variants are best analyzed using SNP-set methods such as gene-set or pathway-based analyses. However, there is as yet no clear consensus regarding how to focus in on potential risk variants following set-based analyses. We used a stepwise, telescoping approach to analyze common- and rare-variant data from the Illumina Metabochip array to assess genomic association with colorectal cancer (CRC) in the Japanese sub-population of the Multiethnic Cohort (676 cases, 7180 controls). We started with pathway analysis of SNPs that are in genes and pathways having known mechanistic roles in colorectal cancer, then focused on genes within the pathways that evidenced association with CRC, and finally assessed individual SNPs within the genes that evidenced association. Pathway SNPs downloaded from the dbSNP database were cross-matched with Metabochip SNPs and analyzed using the logistic kernel machine regression approach (logistic SNP-set kernel-machine association test, or sequence kernel association test; SKAT) and related methods.

**Results:**

The TGF-β and WNT pathways were associated with all CRC, and the WNT pathway was associated with colon cancer. Individual genes demonstrating the strongest associations were *TGFBR2* in the TGF-β pathway and *SMAD7* (which is involved in both the TGF-β and WNT pathways). As partial validation of our approach, a known CRC risk variant in *SMAD7* (in both the TGF-β and WNT pathways: rs11874392) was associated with CRC risk in our data. We also detected two novel candidate CRC risk variants (rs13075948 and rs17025857) in *TGFBR2*, a gene known to be associated with CRC risk.

**Conclusions:**

A stepwise, telescoping approach identified some potentially novel risk variants associated with colorectal cancer, so it may be a useful method for following up on results of set-based SNP analyses. Further work is required to assess the statistical characteristics of the approach, and additional applications should aid in better clarifying its utility.

**Electronic supplementary material:**

The online version of this article (10.1186/s12864-018-4910-8) contains supplementary material, which is available to authorized users.

## Background

In the search to uncover the missing heritability of complex human diseases [1, 2], agnostic analyses of genome-wide SNP array or sequencing data are giving way to SNP-set or gene-set analyses using methods such as burden tests and kernel association tests. Although such combined-locus approaches are well understood, how to proceed to the next step of identifying the individual risk loci is an area that remains open for development. Our aim was to implement existing set-based testing methods and then use the results to focus in on genes and SNPs to seek new candidate risk variants, without imposing rigid testing criteria, in the spirit of relying on independent external replication as the best way of establishing association [3].

Analyses of individual SNPs, especially less-common ones, suffer from lack of power because multiple-testing adjustment is conservative and individual variants may have low frequencies and small-to-moderate effects. It has therefore been suggested that analyses of groups of variants (both common and rare) that contribute to a common mechanism may be more likely to explain common diseases at the population level, with different variants acting (being present) in different individuals [4]. Across the population, then, many variants might contribute to disease risk via a common pathway or cellular network, so in population-based association analyses power can be increased by combining SNPs within genes or pathways, and treating the SNP set, rather than the individual SNP, as the unit of risk [5–9]. This is especially advantageous with rare variants, because their low frequencies render individual-variant approaches unsuitable [10, 11], although due to low population frequency, aggregate analyses with rare variants might still require greater sample sizes than traditional genome-wide analyses of individual common variants [12]. In addition, an alternative to larger sample sizes and dealing with the challenge of multiple comparisons is to incorporate prior information in the selection of SNP sets for study, to exclude likely non-informative (neutral) loci and thereby increase power by reducing dimensionality and the burden of strict multiple-testing adjustment. We undertook such an approach to study how genetic variants based on genotype data from the Metabochip [13] are associated with colorectal cancer (CRC) in the individuals of Japanese ancestry included in the Multiethnic Cohort (MEC), a prospective cohort study including five racial/ethnic populations (White, Latino, African-American, Japanese-American, and Native Hawaiian) conducted in Honolulu and Los Angeles [14]. Although the Metabochip is not a high-density array, it focuses on a number of metabolic pathways—such as those associated with insulin resistance, lipid metabolism, and obesity—that are thought to be involved in CRC etiology.

CRC is an important target for genomic study because it ranks among the top contributors worldwide to cancer incidence and mortality, with substantial differences by ethnic group and involvement of dietary and other lifestyle factors [15–17]. As of 2014, CRC was the third most common cancer and third leading cause of cancer death in both men and women in the U.S. [18], and as of 2012 CRC rates in Miyagi Prefecture, Japan, were the highest among a worldwide selection of registries [16]. Anywhere from 5 to 10% [19] to as much as 15–30% [20] of CRC may be due to known hereditary conditions, including hereditary non-polyposis colorectal cancer (HNPCC; also known as Lynch syndrome, caused by mutations in mismatch DNA repair genes) and familial adenomatous polyposis (FAP, which is caused by mutations in the *APC* tumor suppressor gene). The remainder, sporadic CRC, is commonly attributed to environmental factors, such as a high-caloric, low-fiber, low-calcium western-type diet, low physical activity, obesity, alcohol, and smoking, which presumably involve interactions with predisposing genomic variants [21]. Importantly, offspring of Japanese migrants to Hawaii have had increased rates of CRC far exceeding rates in Japan and even higher than rates in the white population [19, 22]. In recent decades, CRC rates in Japan have increased markedly and have now reached levels that are the same as, or higher than, rates in the United States [23]. Although much of the high incidence of CRC in Japanese is attributed to environmental factors, it is likely that gene-environment interaction also plays a role [24].

The goal of our investigation was to evaluate the use of SNP-set analysis as a preliminary step in ultimately focusing in on potential risk variants. By using what might be called a “telescoping” approach, we began with candidate pathways to limit the initial search for risk variants, then we focused in on genes within the pathways that evidenced association, and finally we zeroed in on variants within the genes that appeared to be associated. Although not a rigorous procedure from the standpoint of statistical testing, such an approach is expected to have greater power to identify potential causal variants than whole-genome testing based on individual-SNP analyses, if it is followed by independent studies focused on the candidate variants.

## Methods

### Study population and genotyping

The MEC, comprising more than 200,000 persons, was assembled in 1993–1996 by the mailing of a self-administered, 26-page questionnaire to persons with drivers licenses (California and Hawaii), voter registrations (Hawaii only), or health care financing records (California only) to obtain extensive information on demographics, medical and reproductive histories, medication use, family history of various cancers, physical activity, and diet. Ancestry in the MEC was ascertained via questionnaire [25]. Because the importance of certain cellular pathways might vary due to ethnic differences, focusing on persons of a single ancestry should be advantageous by reducing variability. We therefore restricted our analysis to persons of Japanese ancestry, for reasons explained in the Background section. The Japanese-American sub-population constitutes about 26% of the MEC.

Identification of incident cancer cases was by regular linkage with the Hawaii, Los Angeles County, and California SEER registries. Although colon and rectal cancers are distinct and have separate ICD codes, they are often combined because their etiologies are similar. In the present analysis we used all CRC and colon cancer only; we did not analyze rectal cancer alone due to the small number of cases.

Genotyping was performed in blood specimens collected according to a case-control design. Some MEC subjects were re-contacted, mostly from 1995 to 2001, for blood collection; these included persons with incident breast, prostate, or colorectal cancers, as well as a random sample of cohort participants to serve as controls in nested genetic case-control studies (participation rate 72% among cases and 63% among controls). From 2001 to 2006, blood was also collected prospectively, without regard to cancer diagnosis, from willing cohort participants (participation rate 43%).

Genotypes were assessed with the Metabochip, a custom Illumina iSelect array designed with about 200,000 SNPs to study genetic association with metabolic, cardiovascular, and anthropometric traits. The Metabochip was not designed to study cancer, but it includes variants known or suspected to be associated with metabolism, obesity, and insulin resistance—factors that have been linked to CRC risk. Although the Metabochip has limited coverage of the genome, larger agnostic GWAS arrays are likely to include large numbers of non-informative SNPs, which can reduce power in gene-set analyses [26]. In addition, although imputation allows estimation of many non-genotyped variants, imputation is challenging with rare variants [26] and imputation with persons of Japanese ancestry in the MEC is based on East Asians in the 1000 Genomes Project [27], which might not be the most suitable basis for imputation of rare variants among persons of strictly Japanese ancestry. We therefore considered that the Metabochip genotype data (without imputation) could be useful for a preliminary examination of CRC pathways because of its focused nature and direct genotyping of less-common variants.

The study protocols of the MEC GWASs were approved by the University of Hawaii Human Studies Program and the University of Southern California IRB.

### Data pre-processing

We chose candidate pathways for the first step (pathway analysis) by assessing published reviews of molecular characteristics of CRC and selecting pathways and their related genes that were anticipated to be associated with CRC, a strategy that is expected to improve power [28, 29]. A similar approach was also described by Liu and others [30]. Molecular characteristics of CRC are the subject of several reviews [20, 31–33]. Pathways chosen were WNT, TGF-beta, P53, RTK-RAS, MAPK, adiponectin, combined DNA repair and fidelity of DNA replication, mTOR, and the laminin gene family. Genes we selected from among these pathways are listed in the Additional file 1. We downloaded lists of SNPs in the selected genes from the dbSNP database [34], similar to the approach of Scarbrough and others [8], except that we did not restrict upstream and downstream distances of candidate variants (it is not clear how association tests with aggregated variants will perform with non-coding variants [12] possibly involved in regulation, so to err on the side of not leaving anything out, we chose to include as many variants as possible). Lists of SNP rs numbers in the selected genes were queried with restriction to “Organism: *Homo sapiens*” and “Variation class: SNP” in the dbSNP database. We copied the list of rs numbers shown in the “dbSNP Batch” option of “Display Settings”. The “dbSNP Batch” list includes rs numbers of SNPs that have been merged with other SNPs, which is important given the time that has elapsed between specification of variants for the Metabochip array and downloading of SNP lists (older, merged rs numbers were not included in the “FlatFile” option of dbSNP).

The downloaded lists of rs numbers were then matched against the rs numbers of variants in the Metabochip data to create the list of variants for analysis (an R script for this processing is available upon request). Downloads were current as of July 28, 2016 or later and were based on genome build 38. Matching SNPs were identified in all pathways except for the laminin gene family. Numbers of SNPs that matched to the Metabochip are shown in the Additional file 1.

We excluded cohort participants whose reported sex did not match the sex chromosome genotype, whose overall genotype call rate was less than 95%, who were first-degree relatives, or whose genotype was found to be duplicated. Of the 8187 remaining participants of Japanese ancestry, 331 were ineligible due to prior cancer, leaving 7856 subjects for analysis: 676 with colorectal cancer (478 with colon cancer) and 7180 controls. We included all genotyped loci from the Metabochip that remained after we excluded markers not in Hardy-Weinberg equilibrium, markers with call rate less than 95%, and sex-chromosome and mitochondrial DNA SNPs. After these variant exclusions there were 189,127 Metabochip SNPs available for matching to the downloaded pathway SNPs. Variants with minor allele frequency (MAF) less than 1% were retained for the present analyses because genes containing common variants with established effects on complex diseases might also contain rare variants with larger effects [1].

### Analysis

To implement the telescoping approach, in the first step we applied the Sequence Kernel Association Test (SKAT [35], formerly known as the logistic SNP-set kernel-machine association test [29]) to pathways as units of analysis. In the second step we applied SKAT to the set of genes within each pathway (one pathway at a time) that showed evidence of association with CRC. In the third step, individual SNPs contained within the genes that evidenced association in analyses with SKAT were analyzed with PLINK [36]. Logistic models (defined below) were fit in SKAT and PLINK with the one-parameter linear genetic effect (count of minor-variant alleles: 0, 1, or 2) as the genomic covariate. *P* values corrected for multiple testing were obtained with the Bonferroni family-wise error rate (FWER) and the false discovery rate (FDR) procedures (note that FDR is not necessarily preferable to FWER in situations with a small number of tests, such as when confirming results in an independent candidate-SNP study [37]). Individual SNPs that showed evidence of association were further examined by searching for their rs numbers in the NHGRI-EBI GWAS Catalogue [38] and in PubMed.

Pathway analyses were performed with the kernel logistic regression procedure in the SKAT R package (v. 1.2.1) [39], which can accommodate rare variants [35]. Briefly, SKAT is based on a variance component score statistic, where the variance component is the variance—within a pathway—of individual-variant effects assumed to follow a common distribution, so that the null hypothesis—that all individual-variant effects are zero—is equivalent to the simpler hypothesis that the variance of those effects is zero. For a binary phenotype (outcome) *Y* ∈ {0,1}, the logistic regression model is

1 $$ \mathrm{logit}\Pr \left({Y}_i=1\right)={\alpha}^{\hbox{'}}{x}_i+{\beta}^{\hbox{'}}{g}_i, $$

where logit is the logistic function {logit(*p*) = log(*p*/[1−*p*])}, the subscript *i* signifies individual *i* in the study population (*i* = 1, …, *n*), ***x***_*i*_ = {1, *x*_*i*1_, …, *x*_*ip*_}′ is the vector of covariates for individual *i*, ***g***_*i*_ = {*g*_*i*1_, …, *g*_*iq*_}′ is the vector of genotypes at *q* SNPs for individual *i*, ***α*** is a (*p* + 1)×1 vector of coefficients for the covariates, and ***β*** is a *q*×1 vector of coefficients for the SNPs. The variance component statistic (*S*) is

$$ S=\left(\mathbf{y}-\widehat{\mathbf{y}}\right)\ {\mathbf{GWG}}^{\hbox{'}}\ \left(\mathbf{y}-\widehat{\mathbf{y}}\right), $$

where **y** = {*y*_1_, …, *y*_*n*_}^'^ is the vector of observed outcomes, $\widehat{\mathbf{y}}$ is the vector of fitted values under the null hypothesis $\left(\hat{\mathbf{y}}={\mathrm{logit}}^{-1}\left[\mathbf{X}\hat{\alpha}\right],\kern0.5em \mathbf{X}={\left[{x}_1,\dots, {x}_n\right]}^{\hbox{'}}\right)$, **G** = [***g***_1_, …, ***g***_*n*_]^'^, and **W** is a *q*×*q* diagonal matrix of individual-variant weights that can be chosen to improve power (e.g., by down-weighting non-functional variants [35]). We used an approach similar to that used by Saunders and others [40], in that we ran alternative analyses with various omnibus tests, such as the optimized combination of burden and SKAT tests (SKAT-O) [41]—which has better power than traditional burden tests [42]—and a test for combined rare and common variants (SKAT-C) [43]. We used the default linear-weighted method in SKAT, which assigns higher weights to rarer variants due to their greater likelihood of being causal.

Covariates we adjusted in logistic regression models were age (*a*), sex (*s*), body mass index (BMI, the Quetelet index *q* = height/weight^2^), smoking behavior (*c*), and, to account for population stratification, the top five ancestry-informative eigenvectors (*p*_1_, …, *p*_5_) from the principal component decomposition of the genotype matrix among MEC Japanese. Logistic regression models for a single variant (*j* ∈ {1, …, *q*}) were therefore of the form

$$ \mathrm{logit}\left[{\Pr}_i\left(\mathrm{cancer}\right)|{x}_i,{g}_{ij}\right]={\alpha}_0+{\alpha}_a{a}_i+{\alpha}_s{s}_i+{\alpha}_q{q}_i+{\alpha}_h{h}_i+{\alpha}_c{c}_i+\sum \limits_{k=1}^5{\alpha}_{p_k}{p}_{ik}+{\beta}_{g_j}{g}_{ij} $$

where Pr_*i*_(cancer) is the probability that individual *i* has colorectal cancer (or colon cancer, depending on which outcome is being analyzed) and *g*_*ij*_ is the *j*th genomic covariate (count of minor alleles) for individual *i*. Logistic models used in SKAT had the obvious multi-locus extension of the genomic covariate, as shown in eq. (1). Height (*h*) was included to remove possible residual dependence of BMI on stature [44], although it had little impact on the results.

SKAT produces a *P* value plot (QQ plot based on the expected uniform distribution of *P* values under the global null hypothesis) as a means of assessing true positives [45]. The *P* values in SKAT are adjusted for poor adherence to asymptotic test assumptions with a binary outcome but are not a priori adjusted for multiple testing (multiple SNP sets).

## Results

### Pathway and individual-gene results

**Fig. 1:**
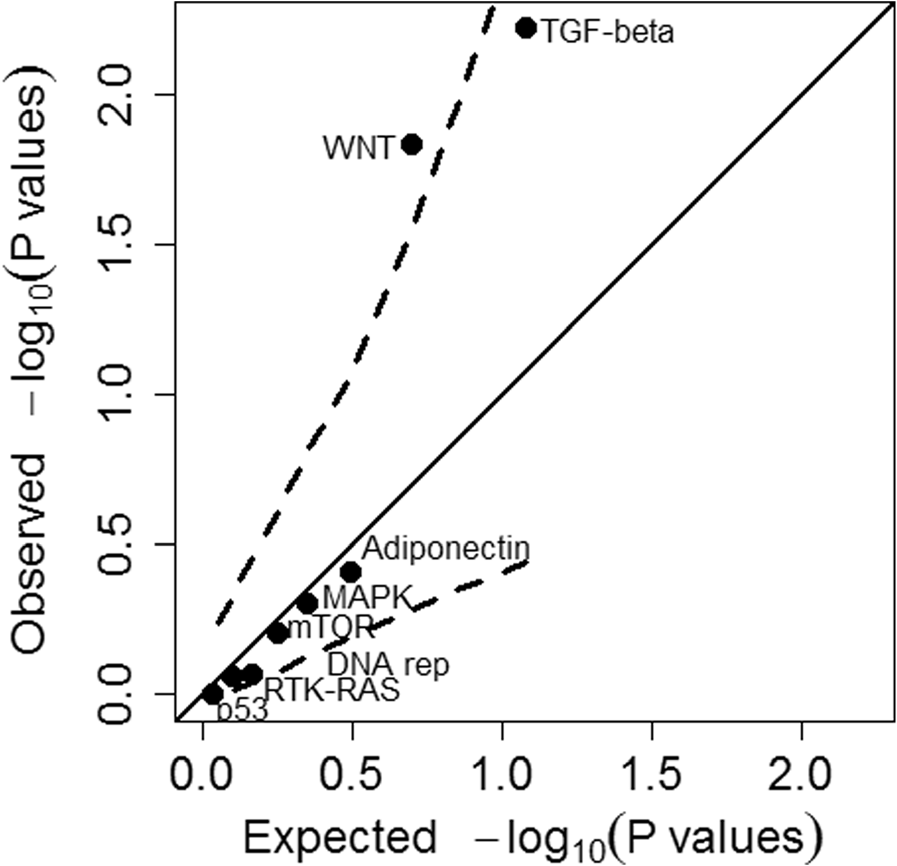
Values of –log10 *P* for individual pathways with adjustment for all covariates. Covariates adjusted were age, sex, BMI, height, smoking behavior, and the top five principal components of the genotype matrix. Solid line: line of identity. Dashed lines: approximate 95% confidence bands (these bands are not precise and should only be used for guidance). Typically, points towards the right that fall well above the line of identity are considered as providing evidence of departure from the null hypothesis of no association

With adjustment for all covariates, the TGF-β (*P* = 0.0060) and WNT (*P* = 0.015) pathways demonstrated associations with all CRC (Fig. 1). With multiple testing adjustment by the Bonferroni and FDR procedures, only the TGF-β pathway evidenced association (Bonferroni *P* = 0.048 based on eight tests, Benjamini & Hochberg FDR P = 0.048 with the R p.adjust [“method = fdr”] procedure; corresponding values for the WNT pathway were 0.12 and 0.058).

With the bootstrap facility in SKAT to adjust for multiple testing, the family-wise error rate adjusted *P* value for TGF-β was 0.087 and that for WNT was 0.17. The SKAT-C method produced unadjusted *P* values 0.052 for WNT and 0.90 for TGF-β (the TGFβ and WNT pathways had similar proportions of common and rare SNPs; see Appendix 3). The SKAT-O combination of traditional-burden and SKAT methods produced *P* values 0.012 for TGF-β and 0.018 for WNT. Given that the TGF-β and WNT pathways are closely related [46], we also performed a pathway analysis with TGF-β and WNT combined as a single pathway; the combined pathway had an unadjusted *P* = 0.0023 (corrected *P* = 0.016 by both Bonferroni and FDR).

Several genes demonstrated evidence of association with CRC when SKAT was applied to genes as SNP sets within either the TGF-β or the WNT pathway (Table 1). None of these genes evidenced association after Bonferroni correction: significance thresholds were 0.05/12 = 0.0042 for TGF-β genes and 0.05/13 = 0.0038 for WNT genes (numbers of genes—12 and 13—that overlapped with the Metabochip were derived from Additional file 1: Table S1). The smallest *P* value was for *WNT11* (based on only one overlapping SNP), whereas the gene with the next smallest *P* value (*TGFBR2*) contained 78 overlapping SNPs.

**Table 1:** Individual genes in the TGF-β and WNT pathways that demonstrated an association with colorectal cancer

Gene	No. SNPs	Pathway	*P* value	
			All CRC	Colon cancer only
*WNT11*	1	*WNT*	0.0064	0.014
*TGFBR2*	78	*TGF-β*	0.0075	0.15
*SMAD7*	6	*TGF-β and WNT*	0.015	0.058
*TCF7L2*	26	*WNT*	0.019	0.045
*TFDP1*	1	*TGF-β*	0.044	0.040

In an analysis with colon cancer only as the endpoint, the WNT pathway appeared to be associated (*P* = 0.045 not corrected for multiple testing) but the TGF-β pathway did not (*P* = 0.18). With the SKAT-C method, *P* = 0.11 for the WNT pathway, and with the SKAT-O method *P* = 0.036 for the WNT pathway. Within the WNT pathway, individual genes evidencing association with colon cancer were *WNT11* (*P* = 0.014) and *TCF7L2* (P = 0.045). *SMAD7* showed only weak evidence of association with colon cancer (*P* = 0.058). The *TFDP1* gene in the TGF-β pathway demonstrated an association with colon cancer even though the TGF-β pathway overall did not. None of these putative associations with colon cancer would be deemed statistically significant after correction for multiple testing, however. For this reason, and because pathway results were qualitatively similar for all CRC and colon cancer only, we used only CRC in further analyses of individual variants.

### Individual-SNP results

Eleven individual Metabochip SNPs in the TGF-β and WNT pathways were associated with CRC in unadjusted case-control association analysis (Table 2; two SNPs—rs11874392 and rs4464148**—**are in both pathways). With covariate adjustment, three SNPs in the TGF-β pathway (rs17025857 and rs13075948 in the *TGFBR2* gene, and rs11874392 in the *SMAD7* gene) were associated with CRC; one of these (rs11874392) is also in the WNT pathway, but no other SNPs in the WNT pathway were associated with CRC after covariate adjustment. With restriction to the 405 Metabochip SNPs in our chosen pathways, the minimum value of FDR was 0.41 (Table 2); Bonferroni adjustment resulted in significance levels of 1.0 for all 405 SNPs.

**Table 2:** Association with colorectal cancer of individual TGF-β and WNT pathway SNPs

SNP	Gene	Minor allele frequency		Crude (unadjusted) association				Adjusted association^a^			
		Cases	Controls	Odds ratio	95% CI	*P* value	FDR^c^	Odds ratio	95% CI	*P* value	FDR
*TGF-β pathway*											
rs17025857	*TGFBR2*	0.029	0.019	1.57	1.12, 2.21	0.009	0.48	2.01	1.28, 3.16	0.002	0.41
rs13075948	*TGFBR2*	0.026	0.018	1.49	1.04, 2.13	0.029	0.58	1.95	1.22, 3.11	0.005	0.44
rs11874392	*SMAD7*	0.375	0.342	1.16	1.03, 1.30	0.015	0.49	1.16	1.00, 1.34	0.049	1
rs3825977	*SMAD3*	0.453	0.490	0.86	0.77, 0.96	0.009	0.48	0.87	0.76, 1.01	0.063	1
rs4776890	*SMAD3*	0.206	0.234	0.85	0.74, 0.97	0.019	0.49	0.85	0.72, 1.01	0.059	1
rs4464148	*SMAD7*	0.038	0.053	0.70	0.53, 0.94	0.016	0.49	0.71	0.50, 1.01	0.057	1
rs11466531	*TGFBR2*	0.011	0.019	0.57	0.34, 0.97	0.034	0.59	0.71	0.39, 1.28	0.26	1
rs3773662	*TGFBR2*	0.011	0.020	0.56	0.33, 0.94	0.026	0.56	0.68	0.38, 1.23	0.20	1
*WNT pathway*											
rs2439593	*APC*	0.0022	0.0003	6.39	1.53, 26.8	0.004	0.48	NA^b^	NA	NA	NA
rs4944092	*WNT11*	0.156	0.129	1.24	1.07, 1.45	0.006	0.48	1.13	0.93, 1.38	0.22	1
rs11874392	*SMAD7*	0.375	0.342	1.16	1.03, 1.30	0.015	0.49	1.16	1.00, 1.34	0.049	1
rs4464148	*SMAD7*	0.038	0.053	0.70	0.53, 0.94	0.016	0.49	0.71	0.50, 1.01	0.057	1
rs11196187	*TCF7L2*	0.023	0.033	0.68	0.47, 0.99	0.042	0.60	0.69	0.43, 1.12	0.13	1

^a^Adjusted for age, sex, BMI, height, smoking status, and the first five principal components among Japanese; odds ratios based on a linear genetic effect

^b^NA: could not be estimated because of low frequency in controls

^c^FDR: false discovery rate based on the 405 Metabochip SNPs that were in the selected pathways

The variant rs11874392 in *SMAD7* (adjusted OR 1.16) was reported by Jiang and others [47] to be associated with CRC in a population-based study of non-Hispanic white subjects, but with odds ratio less than 1 (OR 0.80), whereas it was noted to be positively associated with CRC in Hispanics by Schmit and others (OR = 1.27) [48]. The variant rs13075948 in the *TGFBR2* gene (OR 1.95) is an intron variant that has been implicated in abdominal aortic aneurism [49], but it did not otherwise return any results in searches on NHGRI and PubMed, nor did the variant rs17025857, also an intron variant in the *TGFBR2* gene (adjusted OR 2.01), although other variants in the *TGFBR2* gene have been reported to be associated with CRC [50, 51].

We compared frequencies of the SNPs we detected as being associated with CRC in our population of Japanese ancestry with those of the Tokyo Japanese population (JPT; 120 samples) in the 1000 Genomes Catalog (using the NCBI 1000 Genomes Browser, Phase 3 [52]). All three minor allele frequencies in our population were slightly higher than those in the Tokyo Japanese: SNP rs17025857 (our cohort MAF 0.020) had MAF 0/120 in the Tokyo Japanese; rs13075948 (our cohort MAF 0.019) had MAF 0/120 in the Tokyo Japanese; and rs11874392 (our cohort MAF 0.34) had MAF 0.26 in the Tokyo Japanese.

## Discussion

Agnostic (individual-variant) approaches to association testing can suffer from a lack of statistical power due to low variant frequency and moderate-at-best effect sizes. SNP-set or gene-set analyses (pathway analyses) based on burden tests or kernel association tests are a more powerful approach but do not reveal individual causal SNPs. Recent methodological work has turned to this problem of identifying causal variants after set-based testing, but the area remains open for further development. Based on the premise that the most valid approach to confirming relationships between variants and disease is to conduct independent external replication, a variant-discovery approach that identifies candidates for further, independent investigation should be a useful first stage in identifying risk variants (see Robertson and others [53] for recent work on combining data from two-stage studies). In the present work we illustrate a multi-step, telescoping approach that is motivated not by rigorous significance testing but rather by sequentially removing natural layers of complexity in the analysis. We suggest some variants that might deserve further study in relation to colorectal cancer, but our illustration is not meant to be conclusive with regard to the association of these variants with CRC. The approach can be applied to genome-wide array data or whole-exome (or whole-genome) sequencing data with the intention to follow up results with independent data (including in silico studies).

Our analysis of SNP sets with the SKAT method, which began by limiting the set of candidate SNPs to those in pathways having a known mechanistic role in CRC, identified several variants in the TGF-β and WNT signaling pathways that are potentially associated with CRC in Japanese Americans. Two of those variants (rs17025857, OR = 2.01, and rs13075948, OR 1.95, both in the in *TGFBR2* gene) are apparently new findings given that their association with CRC was not noted in the NHGRI-EBI GWAS Catalog or in PubMed. TGF-β and WNT pathway proteins influence cell division and cell fate of gut endoderm stem cells, such that disorders in these pathways can lead to gastro-intestinal cancers, including colonic adenocarcinomas [46]. Association tests using gene sets within these pathways confirmed that *TGFBR2* in the TGF-β pathway, and *SMAD7* in both pathways, are associated with CRC. Although no individual variants in our analysis would be considered statistically significant based on traditional multiple-testing adjustment methods, the purpose of our analysis was to find previously unidentified candidate risk variants within pathways and genes already known to be related to CRC, so strict *P*-value adjustment—which is conservative and may result in false-negative results (type II errors)—might not be appropriate. Correcting the family-wise error rate assumes a global null hypothesis of no associated elements, whereas it is likely that some, and perhaps many, of the SNPs in the chosen pathways are associated with CRC. By using a stepwise approach, starting with candidate pathways and then telescoping in on genes and then SNPs within genes that demonstrate evidence of association with CRC, some of the overly conservative restrictiveness of traditional multiple testing (and resulting low probability of detecting risk variants) may be overcome. However, such a multi-step approach could still be subject to inflated type I errors, so our findings should only be considered preliminary. As with all genomic analyses, the most important evidence must come from independent confirmation in independent populations. Low density of coverage of the genome might limit the effectiveness of pathway analysis [54], which may be a reason why our selected pathways other than TGF-β and WNT, also known to be associated with CRC, did not show evidence of association in our analysis. Indeed, among our selected pathways, the TGF-β pathway had the largest number of SNPs present on the Metabochip, so it might not be surprising that it produced the strongest evidence of association.

There has been little guidance on how to identify individual driver (risk) SNPs that underlie a SNP set found to be related to phenotype. Various ad hoc approaches have been used; for example, Tang and others [55] chose SNPs that were deemed to be associated by individual-SNP analysis and in genes that were deemed to be associated via SKAT-O analysis. Recently, He and others [9] described a variable-selection method incorporated within the SKAT kernel approach that can be used to suggest which SNPs drive the SNP-set association. In particular, finding a SNP set that evidences association does not distinguish between a few driver SNPs with large effects on the one hand and many driver SNPs with lesser effects on the other.

SNP-set analyses present a number of challenges when rare variants are studied. If rare variants in a particular genetic region are enriched among persons with disease, set-based tests should be more powerful than individual-SNP analyses. However, single variants might not contribute greatly to more powerful SNP-set tests if the number of associated variants in any particular gene-set or SNP-set is small. Furthermore, the optimal approach to testing association with SNP sets depends on many factors, including the true (but unknown) proportion of causal SNPs in the SNP set and their effect magnitudes. We therefore employed several approaches (SKAT, SKAT-C, and SKAT-O), but which is most appropriate cannot be known a priori. Another concern is that, with small samples, the asymptotic distribution used for the SKAT test might be inaccurate. However, this has been said to result in inflated *P* values (or loss of power [56]), in which case small-sample bias would not likely cause false-positive results. It is also possible that variant assignment to a particular gene might be incorrect [57].

Appropriate weighting of individual SNPs within SNP sets would be beneficial if it were feasible. In addition to using biological (functional) information for pathway and gene selection, using such information to weight individual variants in the analysis could reduce the impact of non-informative variants within the selected genes and pathways. We used the default weighting in SKAT, which gives higher weight to rarer variants, but there is surely much residual variation in strength of effect even after accounting for variant frequency. More appropriate weights that take function into account might lead to increased power by down-weighting neutral variants that have little or no impact on cellular processes. However, such functional and annotation information may be advantageous only when it is accurate [2], which remains a problem with online databases. Spencer and others [58] described an alternative, Bayesian, approach that allows incorporation of expert prior functional knowledge.

Several strengths and limitations of the present study deserve consideration. The fact that we were able to reproduce associations with previously reported CRC-related genes provides reassurance about the validity of our approach. A second strength is the design of the MEC study, a prospective cohort study with population based sampling. A third strength is that we focused on one ethnic group: differences in allelic variation among populations can result in reduced power if association tests are not performed in specific populations [59]. A fourth strength is that, by focusing on pathways and genes known to be mechanistically linked to CRC, it is more likely that a true risk variant might be detected, because the genes considered should be enriched with variants related to CRC, and with lower degrees of freedom there is less penalty for multiple testing and hence a lower false-negative rate. One limitation of this study is the relatively small number of cases, which could reduce power. A second limitation is sparse coverage of the genome by the Metabochip for many of the pathways and genes considered in our analysis. There may well be variants in these genes that are associated with CRC but that were not included in the present study because they were not genotyped. A more comprehensive SNP set including imputed variants could be more informative, but as noted in the introduction, imputation with rare variants, especially among persons of Japanese ancestry, may be unreliable. Alternatively, SNP-set analyses based on whole-genome sequence data can be employed to include and potentially discover novel CRC-related genes, as was done by Koboldt and others [60], who combined whole-genome sequencing with selection of known susceptibility genes for prostate cancer. A third limitation is the small set of candidate pathways selected. For example, we did not examine DNA mismatch repair gene defects, which have been linked to HNPCC but are rare in sporadic CRC [20], genes related to the inflammation and innate immunity pathways [61], or genes related to glucose metabolism and its interaction with epithelial-mesenchymal transition [62]. Furthermore, we did not investigate variants in genes or pathways not yet known to be associated with CRC. Better informed decisions as to which pathways to include in the analysis—rather than limiting to pathways with demonstrated associated SNPs—could perhaps further increase the likelihood of detecting novel risk SNPs, because there could be many false negative results among published GWAS studies [63]. However, adding candidate pathways could also increase the proportion of non-informative (neutral) SNPs. A fourth limitation is the lack of a well-defined statistical-testing framework for the telescoping approach we used. Larson and others [64] pointed out that type-I error control has not been well studied for gene-set analyses, and they described corrections to SKAT to adjust for multiple testing when there may be substantial overlap of genes across multiple pathways. However, because of our small number of candidate pathways, there was only modest overlap of genes. Although our approach might suffer from lack of tight type-I error control, it is less likely to reject true causal variants, so it should be useful as a first step in identifying candidate SNPs to be assessed in a second stage of independent validation.

Although we noted potential associations of several variants with CRC, one should keep in mind that a mechanism comprises the collective effects of numerous individual minor variants across the population. It is not finding the individual risk variant per se that is the ultimate goal. Rather, the variants identified should be considered as proxies for the entire mechanism in which they participate, and other variants that impact that mechanism should be considered likely candidates (with effects—and therefore strength of associations—dependent, of course, on their relative functionalities). Indeed, it has been noted that with the explosion of the human population, there are likely to have arisen many rare variants that might play roles in complex disease risk, but with only a few individuals in any particular study sample possessing any one particular variant among them [65]. In this regard, the estimated odds ratios for individual variants may be more informative than their *P* values for association.

## Conclusions

A stepwise, telescoping approach to the analysis of dense genomic data—beginning with pathways, then focusing in on genes within the pathways associated with outcome, and finally assessing individual SNPs within the genes that evidence association with outcome—allowed us to identify several potential novel risk variants not previously associated with CRC in traditional analyses. The procedure is exploratory, so these variants require independent validation as well as consideration of their cellular or regulatory functions before they can be regarded as causal SNPs. Our results are meant merely to demonstrate the potential utility of the stepwise approach to SNP-set analyses; the procedure should identify a greater number of potential associated variants if based on a genome-wide scan. Although multiple-testing implications of the stepwise approach could be complex, this type of approach—coupled with subsequent independent confirmation as well as detailed functional considerations—may be preferable to the agnostic approach typically employed with whole-genome scans. Furthermore, additional novel candidate SNPs might be identified if the initial set of candidate pathways is expanded to include ones that are hypothesized to be associated on the basis of functional or biological knowledge even though they have not previously been established as being associated with CRC (due to low power of agnostic analyses). Future, more in-depth, analyses of CRC risk pathways, genes, and variants should be based on denser coverage of the genome, include a larger number of candidate pathways, investigate differences across ethnic populations, and utilize imputed variants that are imputed with reasonable certainty. Because a large number of neutral loci can dilute statistical power, a potentially fruitful area of future research would be to incorporate functional information to weight pathways, genes, and individual variants according to biological expectations of their relevance to the outcome under study.

## Additional file

Additional file 1: Detailed characterization of genes and pathways used in the analysis. Supplementary Material. Detailed characterization of genes and SNPs used in the analysis. (DOCX 51 kb)

## Abbreviations

BMI: body mass index
CRC: colorectal cancer
MAF: minor allele frequency
MEC: Multiethnic Cohort
SKAT: Sequence Kernel Association Test
SNP: single nucleotide polymorphism
