# Supplementary material for: Stepwise approach to SNP-set analysis illustrated with the Metabochip and colorectal cancer in Japanese Americans of the Multiethnic Cohort
Source: BMC Genomics. 2018 Jul 9;19:524. doi: 10.1186/s12864-018-4910-8 (PMC6038257; doi:10.1186/s12864-018-4910-8)
Supplement: Supplementary file 1 — Detailed characterization of genes and pathways used in the analysis. Supplementary Material. Detailed characterization of genes and SNPs used in the analysis. (DOCX 51 kb) [file 12864_2018_4910_MOESM1_ESM.docx]

**Supplementary material**

Stepwise approach to SNP-set analysis illustrated with the Metabochip and colorectal cancer in Japanese Americans of the Multiethnic Cohort

John Cologne, Lenora Loo, Yurii Shvetsov, Munechika Misumi, Philip Lin, Christopher A. Haiman, Lynne R. Wilkens, Loic Le Marchand

Following are the pathways and genes included in the search of dbSNP, numbers of SNPs found to overlap with the Metabochip, and reasons for including the pathways.

**Table S1**. Characterization of pathways and genes included in the study

| **Pathway / genes** | **Total number of SNPs** | **Number of overlapping SNPs** | **Reasons for inclusion** |
| --- | --- | --- | --- |
| **WNT**  *WNT1*  *WNT2*  *WNT2B* (aka *ST7L*)  *WNT3A*  *WNT5A*  *WNT7A*  *WNT8B*  *WNT9A*  *WNT11*  *APC*  *CTNNB1*  *MYC*  *SMAD7*  *VTI1A*  *TCF7L2*  *CDH1*  *SHROOM2* | **80,456**  412  2,311  2,939  3,061  2,081  3,429  1,167  1,815  1,602  10,639  2,328  590  1,958  18,925  11,851  8,132  7,216 | **67**  0  2  4  1  1  0  0  2  1  3  1  5  6  8  26  7  0 | *APC* tumor suppressor gene mutation is a key step in early carcinogenesis with chromosomal instability (CIN) type of CRC (Colussi et al, 2013)  *APC* is among the most frequently mutated genes in non-hypermutated CRC (Cancer Genome Atlas Network, 2012)  WNT signaling pathway is altered in 93% of CRC (Cancer Genome Atlas Network, 2012) |
| **TGF-β**  *TGFB1*  *TGFBR1*  *TGFBR2*  *ACVR2A*  *ACVR1B*  *SMAD2*  *SMAD3*  *SMAD4*  *SMAD7*  *TFDP1*  *E2F4*  *P107*  *E2F5*  *ARID1A*  *MYC*  *BMP2*  *BMP4*  *GREM1* | **53,895**  3,054  2,814  4,884  3,968  2,374  5,308  6,978  2,895  1,958  4,119  814  5,036  1,860  4,811  590  806  574  1052 | **220**  7  12  78  6  0  2  92  8  6  1  0  0  1  2  5  0  0  0 | *SMAD2* and *SMAD4* are on chromosome *18q*, where LOH is associated with negative prognosis with colon cancer (in particular with high metastatic potential in chromosomal instability type; Colussi et al, 2013)  TGF-β receptor II (*TGFBR2*) mutations occur in more than 80% of microsatellite instability CRC, with a switch to tumor-promoting role of TGF-β signaling facilitated by SMAD4 (Colussi et al, 2013)  *SMAD4* is among the most frequently mutated genes in non-hypermutated CRC and TGF-β signaling pathway known to be deregulated in CRC (Cancer Genome Atlas Network, 2012)  *ACVR2* mutations occur frequently in combination with *TGFBR2* mutations |
| **P53**  *ATM*  *TP53* | **10,851**  9,162  1,689 | **4**  0  4 | *p53* loss of function occurs often in later stages of CRC tumorigenesis (simulates proliferation; Colussi et al, 2013)  *TP53* is among the most frequently mutated genes in non-hypermutated CRC (Cancer Genome Atlas Network, 2012) |
| **P13K & RTK-RAS**  *IGF2*  *IGF1R*  *IRS2*  *PIK3CA*  *PIK3R1*  *PTEN*  *ERBB2*  *ERBB3*  *NRAS*  *KRAS*  *BRAF* | **54,682**  1,530  18,914  2,149  4,299  4,506  5,408  2,826  2,078  799  2,372  9,801 | **34**  1  14  3  0  8  2  0  2  2  1  1 | *K-ras* is involved in transition from benign to malignant stages of CRC, and BRAF mutations are frequently associated with poorer CRC prognosis (Colussi et al, 2013)  *KRAS* and *NRAS* among most frequently mutated genes in non-hypermutated CRC (Cancer Genome Atlas Network, 2012) |
| **MAPK**  *PDGFA*  *EGF*  *FGF1*  *PDGFRA*  *PDGFRB*  *EGFR*  *FGFR1*  *FGFR2*  *FGFR3*  *PTPN11*  *MYC*  *DUSP10*  *MYO1B*  *CCND2*  *SH2B* | **75,360**  1,762  5,544  5,933  4,029  3,178  11,382  3,691  7,203  2,162  4,031  590  2,214  8,979  1,894  12,768 | **62**  0  1  7  1  4  8  3  12  1  5  5  0  4  3  8 | High WNT activity is connected with increased MAPK signaling (Colussi et al, 2013) |
| **ADIPONECTIC**  *ADIPOQ*  *ADIPOR1*  *ADIPOR2*  *AMPK* (aka *PRKAA1*/*2*)  *SREBF1* | **31,185**  4,262  4,462  4,717  7,561  10,183 | **15**  8  1  3  3  0 | A tag SNP in the *ADIPOQ* gene flanking region was associated with decreased CRC risk (Kaklamani et al, 2008) [note that an attempt to replicate this was unsuccessful in a UK study; Carvajal-Carmona et al, 2010]  Systemic adiponectin and local adinopectin receptor expression in the colon may be associated with anti-tumorigenesis in early stages of CRC (Tae et al, 2014)  Certain *ADIPOQ* SNPs (rs2241766 T>G, rs1501299 G>T, and rs266729 C>G) are associated with increased risk of CRC (Yang et al, 2015) |
| **Laminin family**  *LAMA5*  *LAMC1* | **32,742**  26,271  6,471 | **0**  0  0 | A novel CRC pathway involved in cell adhesion (Peters et al, 2015)  This pathway was not included in the analysis because there were no variants that overlapped with the Metabochip |
| **DNA repair — fidelity of DNA replication**  *PITX1*  *PITX2*  *PITX3*  *POLD3*  *CDKN1A* (aka *PAK3*)  *FEN1* (aka *MIR611*,*TMEM258*)  *MLH1*  *CHEK2* | **23,804**  579  1,413  704  3,753  9,721  511  3,887  3,236 | **18**  0  0  0  4  9  2  3  0 | One of the established CRC pathways (Peters et al, 2015) |
| **mTOR**  *STK11*  *PTEN*  *AMPK* (aka *PRKAA1*/*2*)  *AKT1* | **17,023**  2,030  5,408  7,561  2,024 | **6**  0  2  3  1 | STK11 and PTEN activities converge in the mTOR pathway and are related to rare hereditary colorectal syndromes (Peters et al, 2015)  The mTOR pathway is related to tumor formation and angiogenesis, insulin resistance, and adipogenesis; it is also intimately tied to the WNT pathway (Laplante and Sabatini, 2009) |

Total overlap of all downloaded SNPs and Metabochip is 0.12% (sum of column 3 divided by sum of column 2).

The number of dbSNP SNPs that overlap with the Metabochip tends to be larger in genes and pathways that contain larger numbers of SNPs (Figure S1). Note that the scales of abscissa and ordinate differ by 2 orders of magnitude and most of the points are near the X axis, which means that far fewer than 1% of the dbSNP listed variants are on the Metabochip. The fact that the TGF-β pathway was by far the best represented on the Metabochip—among the pathways selected—is reflected in the high position on the plot of the point for the TGF-β pathway.

**Figure S1**. Overlap of pathway SNPs with Metabochip SNPs. Open circles show the relationship between total number of SNPs per gene in the dbSNP database (abscissa) and number of those SNPs that matched the Metabochip (ordinate). Closed circles are summaries by pathway (abscissa: mean number of SNPs per gene averaged over all selected genes within each pathway; ordinate: total number of SNPs in each pathway that matched the Metabochip).

Following are the average minor allele frequencies (MAFs) and total number of SNPs (N, in parentheses) by pathway, according to whether SNPs were common (MAF ≥ 0.05), less common (0.01 ≤ MAF < .05), or rare (MAF < 0.01):

| **Pathway** | **Common SNPs** | **Less common SNPs** | **Rare SNPs** |
| --- | --- | --- | --- |
| Adiponectin | 0.34 (10) | 0.021 (1) | 0.00078 (4) |
| DNA replication & fidelity | 0.32 (11) | 0.016 (1) | 0.00052 (6) |
| MAPK | 0.29 (36) | 0.040 (3) | 0.0016 (23) |
| mTOR | 0.40 (2) | 0.021 (1) | 0.0033 (3) |
| p53 | 0.24 (3) | 0.021 (1) | - (0) |
| RTK-RAS | 0.26 (27) | 0.034 (2) | 0.0050 (5) |
| TGF-β | 0.31 (172) | 0.026 (9) | 0.0013 (39) |
| WNT | 0.23 (47) | 0.040 (10) | 0.0012 (10) |

**References**

The Cancer Genome Atlas Network. Comprehensive molecular characterization of human colon and rectal cancer. *Nature* 2012;487(7407):330−337. doi: 10.1038/nature11252

Carvajal-Carmona LG, Spain S, The CORGI Consortium, Kerr D, Houlston R, Cazier J-B, et al. Common variation at the adiponectin locus is not associated with colorectal cancer risk in the UK. *Hum Mol Genet* 2009;18(10):1889−1892. doi: 10.1093/hmg/ddp109

Colussi D, Brandi G, Bazzoli F, Ricciardiello L. Molecular pathways involved in colorectal cancer: implications for disease behavior and prevention. *Int J Mol Sci* 2013;14(8):16365−16385. doi: 10.3390/ijms140816365

Kaklamani VG, Wisinski KB, Sadim M, Gulden C, Do A, Offit K, et al. Variants of the adiponectin (*ADIPOQ*) and adiponectin receptor 1 (*ADIPOR1*) genes and colorectal cancer risk. *JAMA* 2008;300(13):1523–1531.

Laplante M, Sabatini DM. mTOR signaling at a glance. *J Cell Sci* 122:(20)3589–3594. doi: 10.1242/jcs.051011

Peters U, Bien S, Zubair N. Genetic architecture of colorectal cancer. *Gut* 2015;64(10):1623–1636. doi: 10.1136/gutjnl-2013-306705

Tae CH, Kim S-E, Jung S-A, Joo Y-H, Shim K-N, Jung H-K, et al. Involvement of adiponectin in early stage of colorectal carcinogenesis. *BMC Cancer* 2014;14:811. doi: 10.1186/1471-2407-14-811

Yang X, Li J, Cai W, Yang Q, Lu Z, Yu J, et al. Adiponectin gene polymorphisms are associated with increased risk of colorectal cancer. *Med Sci Monit* 2015; 21:2595−606. doi: 10.12659/MSM.893472
